# Application of an Ultrasonic Nebulizer Closet in the Disinfection of Textiles and Footwear

**DOI:** 10.3390/ijerph191710472

**Published:** 2022-08-23

**Authors:** Tiago M. Henriques, Beatriz Rito, Diogo N. Proença, Paula V. Morais

**Affiliations:** 1 UCCCB—University of Coimbra Bacteria Culture Collection, Department of Life Science, University of Coimbra, 3000-456 Coimbra, Portugal; 2 IATV—Instituto do Ambiente Tecnologia e Vida, 3030-790 Coimbra, Portugal; 3 University of Coimbra, Centre for Mechanical Engineering, Materials and Processes, Department of Life Sciences, 3000-456 Coimbra, Portugal

**Keywords:** disinfection, pathogen transmission control, textiles, footwear, ultrasonic nebulization closet, aerosol, bacterial spores, bacteriophage, Gram-positive bacteria, Gram-negative bacteria

## Abstract

The emergence of the coronavirus disease 2019 (COVID-19) pandemic highlighted the importance of disinfection processes in health safety. Textiles and footwear have been identified as vectors for spreading infections. Therefore, their disinfection can be crucial to controlling pathogens’ dissemination. The present work aimed to evaluate the effectiveness of a commercial disinfectant aerosolized by an ultrasonic nebulizer closet as an effective method for disinfecting textiles and footwear. The disinfection was evaluated in three steps: suspension tests; nebulization in a 0.08 m^3^ closet; nebulization in the upscaled 0.58 m^3^ closet. The disinfection process of textiles and footwear was followed by the use of bacteriophages, bacterial spores, and bacterial cells. The disinfection in the 0.58 m^3^ closet was efficient for textiles (4 log reduction) when bacteriophage *Lambda*, *Pseudomonas aeruginosa,* and *Bacillus subtilis* were used. The footwear disinfection was achieved (4 log reduction) in the 0.08 m^3^ closet for *Escherichia coli* and *Staphylococcus aureus*. Disinfection in an ultrasonic nebulization closet has advantages such as being quick, not wetting, being efficient on porous surfaces, and is performed at room temperature. Ultrasonic nebulization disinfection in a closet proves to be useful in clothing and footwear stores to prevent pathogen transmission by the items’ widespread handling.

## 1. Introduction

The emergence of the coronavirus disease 2019 (COVID-19) pandemic caused by the severe acute respiratory syndrome coronavirus 2 (SARS-CoV-2) highlighted the importance of disinfection processes in health safety. Moreover, the risk of the emergence of new variants of SARS-CoV-2 and new pandemics [1], along with the growing problem of multidrug-resistant microorganisms [2], makes the development of new and better disinfection methods necessary.

Manually executed disinfection processes are operator-dependent and prone to failure [3]. Furthermore, many of the contaminated areas are not accessible by conventional manual disinfection methods. On the other hand, automatic disinfection using ultraviolet radiation is not efficient on porous surfaces [4]. Thus, ultrasonic disinfectant nebulization is an automated, easy-to-use alternative that, owing to the small size of the aerosol particles generated, has a high penetration into contaminated areas with difficult access [5]. Moreover, ultrasonic nebulization has advantages over conventional mechanical nebulization methods, such as pressure- or gas-assisted systems. Firstly, the disinfectant aerosol droplets generated by the ultrasonic system are smaller in size. Second, as the ultrasonic system operates at ambient pressure and does not need high speed to produce the nebulization, the ultrasonic generation of the aerosol does not require as much direct mechanical energy as conventional nebulization systems [6].

Textiles, such as clothes or household linens, and footwear, such as shoes or slippers, have been identified by several studies as vectors for spreading infections [7,8,9,10,11]. Indeed, many microorganisms are transferred to textiles and footwear through contact with skin and body excretions. On the other hand, microorganisms can also be transferred from the environment through textiles and footwear contact with, for example, dust, soil, furniture, or food [7,9,10]. Most microorganisms present in textiles and footwear do not pose a health risk since many are part of skin microbiota [7]. However, the contact of textiles and footwear with an infected person or a contaminated environment causes these materials to carry pathogens, thus making them act as fomites and pose a risk to public health [7,8,9,10,11]. Effectively, in healthcare facilities such as hospitals, pathogens are found on hospital linens and on the clothing of both patients and healthcare professionals [7,8]. In the context of the COVID-19 pandemic, Aumeran et al. [12] detected the presence of SARS-CoV-2 on the gowns of healthcare workers who treated COVID-19 patients, and Redmond et al. [13] detected the presence of SARS-CoV-2 on personnel shoes of a COVID-19 ward. Furthermore, pathogens can remain viable in textiles and footwear for long periods of time [14,15,16,17,18,19,20,21,22]. At room temperature, Owen et al. [15] showed that HCoV-OC43 remains infectious on polyester, cotton, and polycotton for at least 72 h, 24 h, and 6 h, respectively, and HCoV-229E remains infectious on polyester, cotton, and polycotton for at least 24 h, 2 h, and 2 h, respectively. For their part, Shivkumar et al. [16] detected HCoV-OC43 for 6 h, 24 h, and 48 h on patent leather, finished leather, and calf leather, respectively. In another study, Harbourt et al. [18] investigated the stability of SARS-CoV-2 in clothing at different temperatures and 40–50% relative humidity. Despite not detecting viable viruses at 37 °C after initial exposure, the authors found that at 4 °C the virus remained viable for at least 96 h and that at 22 °C the virus remained viable for at least 4 h. In turn, Chin et al. [19] investigated the stability of SARS-CoV-2 on cloth and surgical masks at room temperature with a relative humidity of around 65%. These authors found infectious viruses being detectable on the cloth after 1 day, on the inner mask layer after 4 days, and on the outer mask layer after 7 days [19]. For their part, Fijan et al. [20] found that *Enterococcus faecium*, *Staphylococcus aureus*, and *Pseudomonas aeruginosa* survive several days on cotton textile swatches at 25 °C. Riley et al. [21] also found that *Escherichia coli* and *S. aureus* survive several days in cotton and polyester textile swatches at 23 °C and 47% relative humidity. In another study, Hanczvikkel and Tóth [22] found several species of multidrug-resistant bacteria that survive several days on a cotton towel at 25 °C and 52% relative humidity. Therefore, laundering is usually employed in textiles and footwear, not only to clean but also to disinfect [7,8,9,10,11,23,24,25]. Nevertheless, the washing machine itself can be a source of contamination for textiles and footwear [7,10,26]. In this case, contamination can occur due to biofilm formation inside the washing machine, which can work as a reservoir of pathogens, or cross-contamination between materials, which can occur when a contaminated item is put in the washing machine along with uncontaminated laundry items [7]. In addition to possibly contaminating clothes and footwear, laundering is also a time-consuming process that requires drying. Therefore, laundering is not a practical process for disinfecting clothes and footwear in contexts where rapid disinfection without wetting is required. One such context is the disinfection of clothing and footwear in stores after customer handling.

Some studies have investigated the disinfection of textiles and footwear through coarse spray disinfectant application [27,28,29,30,31,32,33,34,35,36]. However, few studies have explored the disinfection of textiles and footwear through fine aerosol disinfectant application inside a disinfection chamber. Recently, as part of fighting the spread of COVID-19, Abu-Zidan et al. [37] proposed a prefabricated portable chamber that sprays individuals with fine mist sanitizing fluid to disinfect the clothing and exposed surfaces of people. For their part, Khan et al. [38] studied a solar-powered disinfection tunnel mist spray to disinfect individuals’ clothing in large gatherings. Previously, in order to disinfect various surfaces, including synthetic-fiber carpets and cotton fabric, Callahan et al. [39] developed a chamber for the application of a nebulized disinfectant. In addition, some patents also describe apparatus for ultrasonic nebulization of disinfectants [40,41,42,43,44,45]. Nevertheless, to the best of our knowledge, the use of an ultrasonic nebulizer closet for the disinfection of textiles and footwear has never been studied. Therefore, the present work aimed to evaluate the effectiveness of a commercial disinfectant aerosolized by an ultrasonic nebulizer closet as an effective method to quickly disinfect textiles and footwear after customers handle it in stores. The disinfection process was followed by the use of *Bacillus atrophaeus* DSM 2277 spores as an indicator of chemical sterilization efficiency, bacteriophage *Escherichia virus Lambda* DSM 4499 as an indicator of virucidal activity, and vegetative cells of *E. coli* DSM 30083, *P. aeruginosa* DSM 1117, *S. aureus* DSM 20231, and *Bacillus subtilis* DSM 10 as indicators of bactericidal activity.

## 2. Materials and Methods

### 2.1. Microbiological Indicators Production and Preservation

*B. atrophaeus* DSM 2277, bacteriophage *Lambda* DSM 4499, *E. coli* DSM 4230 (bacteriophage *Lambda* host), *E. coli* DSM 30083, *P. aeruginosa* DSM 1117, *S. aureus* DSM 20231, and *B. subtilis* DSM 10 were obtained from the Leibniz Institute DSMZ-German Collection of Microorganisms and Cell Cultures GmbH, Braunschweig, Germany. Bacteria were grown on agar medium plates according to the incubation conditions shown in Table 1 and preserved at −80 °C in nutrient broth (NB) medium supplemented with 15% (*v*/*v*) glycerol.

For bacteriophage *Lambda* DSM 4499 multiplication, the host bacterium *E. coli* DSM 4230 was plated on Luria–Bertani (LB) agar medium through the top agar layer method. For that, 100 μL of host bacterium *E. coli* DSM 4230 culture in 0.85% (*w*/*v*) NaCl suspension with 0.3 optical density (OD) at 600 nm was mixed with 5 mL of LB soft agar medium (0.75% (*w*/*v*) agar) at 50 °C and plated over LB agar medium. A filter paper containing the dried bacteriophage suspension was placed in the center of the host plate, 100 μL of LB broth medium was added to the surface, and the plate was incubated overnight under the conditions of the host bacterium (37 °C). After incubation, a halo was visible around the filter paper, which resulted from the lysis of the host cells. To prepare a bacteriophage stock suspension, 5 mL of LB broth medium was added to the plate and then placed on a slowly rotating shaker (GFL^®^ 3005, GFL Gesellschaft für Labortechnik mbH, Burgwedel, Germany) at room temperature for at least 4 h. After this period, the bacteriophage suspension was centrifuged at 5000× *g* for 20 min at 4 °C (VWR^®^ MicroStar 17R, VWR International BV, Leuven, Belgium). The resulting supernatant was filtrated with a sterile cellulose acetate syringe filter of 0.45 μm porous size (VWR International LLC., Radnor, PA, USA) to remove the remaining bacteria, and the filtrate (bacteriophage stock suspension) was stored at 4 °C.

To obtain spores of *B. atrophaeus* DSM 2277, cells were inoculated in a 300 mL Erlenmeyer flask containing 120 mL of NB medium and incubated at 130 rpm for 3 days at 30 °C in an orbital shaker (S200D, Comecta S.A., Barcelona, Spain). After growth, cells were harvested by centrifugation at 8801× *g* for 30 min at 4 °C and resuspended in 15 mL. The cell suspension was pasteurized at 85 °C for 15 min and placed on ice for more than 15 min. The suspension was passed through a high-pressure homogenizer (EmulsiFlex^®^ C3, Avestin Inc., Ottawa, ON, Canada) (15,000–20,000 psi) for 1 min. The lysate was concentrated using Vivaspin^®^ 6 centrifugal concentrator with a molecular weight cut-off of 100 kDa (Sartorius Stedim Biotech GmbH, Göttingen, Germany) at 3150 g for 30 min at 4 °C (Centrifuge 5810R, Eppendorf AG, Hamburg, Germany). This stock spore suspension was stored at 4 °C.

Inocula of *B. atrophaeus* DSM 2277 spores and bacteriophage *Lambda* DSM 4499 were obtained from the respective stock suspensions. Inocula of *E. coli* DSM 30083, *P. aeruginosa* DSM 1117, *S. aureus* DSM 20231, and *B. subtilis* DSM 10 were obtained from vegetative cell suspensions prepared in 5 mL sterile 0.85% (*w*/*v*) NaCl. The inoculum turbidity of the microbiological indicator suspensions was adjusted to five on the McFarland scale.

### 2.2. Disinfectant and Ultrasonic Nebulizer Closets

VIRCOV BAC 360 (Inokem S.A., Forte da Casa, Portugal) is a disinfectant formed by three biocidal active substances: benzalkonium chloride (BAC), which is a quaternary ammonium compound, glycolic acid, and ethanol. All these three substances are included in Article 95 List of European Chemicals Agency (ECHA) for industrial disinfection [46]. Additionally, VIRCOV BAC 360 also contains tetrasodium glutamate diacetate, which acts as a chelating and wetting agent.

Nebulization disinfection tests were carried out in a 0.08 m^3^ closet of 605 mm (height) × 420 mm (width) × 325 mm (length) (Supplementary Materials Figure S1) and in a 0.58 m^3^ closet of 1600 mm (height) × 600 mm (width) × 600 mm (length) (Supplementary Materials Figure S2). The 0.58 m^3^ closet was equipped with a dehumidifier, a heater, and an exhaust fan with activated carbon filters. During the nebulization disinfection tests, the dehumidifier was off, the temperature was between 35 °C and 40 °C, and the exhaust fan was only turned on in the final stage of disinfection tests to remove the aerosol inside the closet through the activated carbon filters. This 0.58 m^3^ closet consisted of a closet scale up and was a prototype of NovirBox from Dynasys—Engenharia e Telecomunicações, S.A., Setúbal, Portugal.

In both closets, nebulization of the disinfectant solution was carried out by a piezoelectric ultrasonic nebulizer. Briefly, the nebulizer had three piezoelectric transducers at the bottom of the disinfectant solution container that converted electrical energy into high-frequency mechanical vibrations. These vibrations caused the liquid above the piezoelectric transducers to be rarefied and compressed at a high-frequency cycle, which led to the formation of a micron-size aerosol from the disinfectant solution [6]. The aerosol formed was conducted into the disinfection closet by a forced draft fan installed at the top of the disinfectant solution container. All nebulization disinfection tests were performed at a nebulization flow rate of 10.4 ± 1.4 mL·min^−1^.

### 2.3. Experimental Design

The disinfection was evaluated in three steps: (1) suspension tests; (2) nebulization tests in a 0.08 m^3^ closet; (3) nebulization tests in the upscaled 0.58 m^3^ closet.

Evaluation of VIRCOV BAC 360 sporicidal and virucidal activity was performed by suspension test, in a 48-well microplate, at 100% concentration and serial 2-fold dilutions in sterile deionized water up to 1/16 (*v*/*v*) dilution. The control was performed with sterile deionized water without disinfectant. *B. atrophaeus* DSM 2277 spores and bacteriophage *Lambda* DSM 4499 were used as indicators with a contact time of 2 min. The suspension tests were carried out in 100 μL of the respective disinfectant dilution inoculated with 5 μL of the indicator suspension.

To evaluate nebulization disinfection effectiveness in both closets, fabric swatches (ca. 6 cm^2^) composed of 88% polyester and 12% elastane (Supplementary Materials Figure S3) were inoculated with 30 μL of each indicator suspension.

In the 0.08 m^3^ closet, the fabric swatches were placed at the bottom of the chamber and subjected to 2 min disinfectant nebulization. Afterward, the fabric swatches were removed 5 min after stopping the nebulization and analyzed. The disinfectant dilutions of 1/2 (*v*/*v*) and 1/3 (*v*/*v*) were used to evaluate the elimination of *B. atrophaeus* DSM 2277 spores and bacteriophage *Lambda* DSM 4499. In the 0.08 m^3^ closet, the disinfection of a folded cotton towel of 460 × 720 mm and of a footwear item was also tested. For that, fabric swatches (ca. 6 cm^2^) inoculated with 30 μL of each indicator suspension were placed inside the folded towel (Supplementary Materials Figure S4) and glass slides (24 × 50 mm) were placed inside the footwear item and then also inoculated with 30 μL of each indicator suspension. In these experiments, the disinfectant dilution of 1/3 (*v*/*v*) was used to evaluate the elimination of *B. atrophaeus* DSM 2277 spores, bacteriophage *Lambda* DSM 4499, *E. coli* DSM 30083, and *S. aureus* DSM 20231. The folded towel and the footwear item were also subjected to 2 min disinfectant nebulization. Five minutes after stopping the nebulization, the fabric swatches and the glass slides were taken and utilized to evaluate the presence of the indicators.

To evaluate the nebulization disinfection effectiveness in the upscaled 0.58 m^3^ closet, the disinfectant was used at 1/3 (*v*/*v*) dilution and the indicators were *B. atrophaeus* DSM 2277 spores, bacteriophage *Lambda* DSM 4499, *E. coli* DSM 30083, *P. aeruginosa* DSM 1117, *S. aureus* DSM 20231, and *B. subtilis* DSM 10. The fabric swatches were placed in the middle of the chamber over a wire mesh. In this closet, a short disinfection cycle and a long disinfection cycle were tested. The short disinfection cycle consisted of 2 min of nebulization, 2 min of rest without nebulization, and 2 min of aerosol extraction from the closet. The long disinfection cycle consisted of 4 min of nebulization, 4 min of rest without nebulization, and 2 min of aerosol extraction from the closet.

### 2.4. Determination of Cell Survival

In suspension disinfection experiments, after the 2 min contact time, serial 10-fold dilutions in sterile 0.85% (*w*/*v*) NaCl were performed and 100 μL were spread on agar plates for colony-forming unit (CFU) enumeration, or in the case of bacteriophages, plaque-forming units (PFU). The plates were incubated according to the conditions shown in Table 1.

In nebulization disinfection experiments, the materials were placed inside the closet, the chamber was closed, and the disinfection cycle proceeded. Immediately after the disinfection cycle was completed, the fabric swatches or the glass slides were placed into 50 mL tubes with 5 mL of sterile 0.85% (*w*/*v*) NaCl, shaken manually, and allowed to incubate for 1 h at room temperature. Serial 10-fold dilutions were made and 100 μL plated for CFU or PFU quantification. The plates were incubated according to the conditions shown in Table 1.

PFU quantification was performed using the 100 μL of the 10-fold serial dilutions mixed with 100 μL of host suspension (0.3 OD_600nm_ in 0.85% (*w*/*v*) NaCl) *E. coli* DSM 4230. After blending the 10-fold serial dilution with the host bacteria, the 200 μL was plated through the top agar layer method, i.e., the 200 μL blend was mixed with 5 mL of LB soft agar medium (0.75% (*w*/*v*) agar) at 50 °C and immediately poured over an LB agar medium plate. Plates were incubated at 37 °C for 24 h. Bacteriophage plaques were counted and calculated to PFU·mL^−1^.

In the nebulization experiments, the control tests were performed by transferring the 30 μL of indicator suspension directly into the 50 mL tubes with 5 mL of sterile 0.85% (*w*/*v*) NaCl.

### 2.5. Data Analysis

In all experiments, the tests with disinfectant were compared to the control tests. The results of the comparison were expressed as log_10_ reduction and elimination rate (%). The log_10_ reduction was calculated through Equation (1):

log_10_ reduction = log_10_(*N*_0_) − log_10_(*N*_1_) (1)

where *N*_0_ is the mean of the CFU·mL^−1^ or PFU·mL^−1^ replicate values of the control test in a given experiment, and *N*_1_ is the CFU·mL^−1^ or PFU·mL^−1^ value of one of the replicates of the disinfection test of a given experiment. For its part, the elimination rate was calculated through Equation (2):

(2) $$\mathrm{Elimination}\;\mathrm{rate}\;\left(\%\right)=\frac{N_{0}-{\;N}_{1}}{N_{0}}\;\times \;100$$

Results were presented as mean ± standard deviation and the number of replicates performed in each test is indicated in the respective figure caption.

Statistical differences between groups were evaluated by the employment of one-way or two-way analysis of variance (ANOVA) where appropriate. After ANOVAs, post hoc comparisons were made by applying the Tukey test. Differences were considered statistically significant when the associated *p*-values were lower than 0.05. The caption of each figure indicates which statistical test was used. Statistical analysis was performed by use of GraphPad Prism 9 for Windows 64-bit, version 9.3.1 (GraphPad Software Inc., San Diego, CA, USA).

### 2.6. Criteria for Disinfection Acceptance

The present study follows the disinfection acceptance criteria presented by ECHA in Guidance on the Biocidal Products Regulation: Volume II Efficacy—Assessment and Evaluation (Parts B + C) [47]. This guide Appendix 4 of [47] presents the available standards for testing the efficiency of biocides when applied in the disinfection of textiles.These standards include suspension tests and carrier tests Appendix 4 of [47]. In suspension tests, the standards EN 13727 (medical applications) [48] and EN 1276 (non-medical applications) [49] are identified for bacterial disinfection, and the standard EN 14476 (medical applications) [50] is identified for virus disinfection. Both EN 13727 and EN 1276 require 5 log_10_ reduction of vegetative bacteria as a pass criterion [47]. For its part, EN 14476 only requires 4 log_10_ reduction of viruses as a pass criterion [47]. Likewise, the guideline of the German Association for the Control of Virus Diseases (DVV) and the Robert Koch Institute (RKI) also requires 4 log_10_ reduction of viruses as a pass criterion for suspension tests [51]. In carrier tests, Appendix 4 of the ECHA’s guidance [47] identifies the standards EN 16616 [52], ASTM E2406 [53], and ASTM E2274 [54] for both bacterial and virus disinfection. For vegetative bacteria, the standards EN 16616, ASTM E2406, and ASTM E2274 require, respectively, 7, 4, and 4 log_10_ reduction as pass criteria [47]. Nevertheless, for viruses, these three standards do not define any pass criterion [47]. Furthermore, none of the standards for textile disinfection mentioned in Appendix 4 of the ECHA’s guidance provide criteria for bacterial spore disinfection [47]. Therefore, in the criteria for disinfection acceptance of the present study, the EN 13704 standard [55] was also considered because this standard provides a quantitative suspension test for the evaluation of the bacterial sporicidal activity of chemical disinfectants used in food, industrial, domestic, and institutional sectors [56]. As a pass criterion, EN 13704 requires a 3 log_10_ reduction of bacterial spores [56].

In the present study, in addition to the criteria mentioned above, the disinfection acceptance criteria presented in the U.S. Pharmacopeia [57] were also considered. According to the U.S. Pharmacopeia, a coupon surface disinfection process (no materials specified) is considered effective if it allows a log_10_ reduction of at least 2 for bacterial spores and 3 for vegetative bacterial cells [57]. However, for viruses, the U.S. Pharmacopeia does not clarify the log_10_ reduction value required for the disinfectant to be considered effective [57].

## 3. Results

### 3.1. Disinfection by Suspension Test

**Figure 1:**
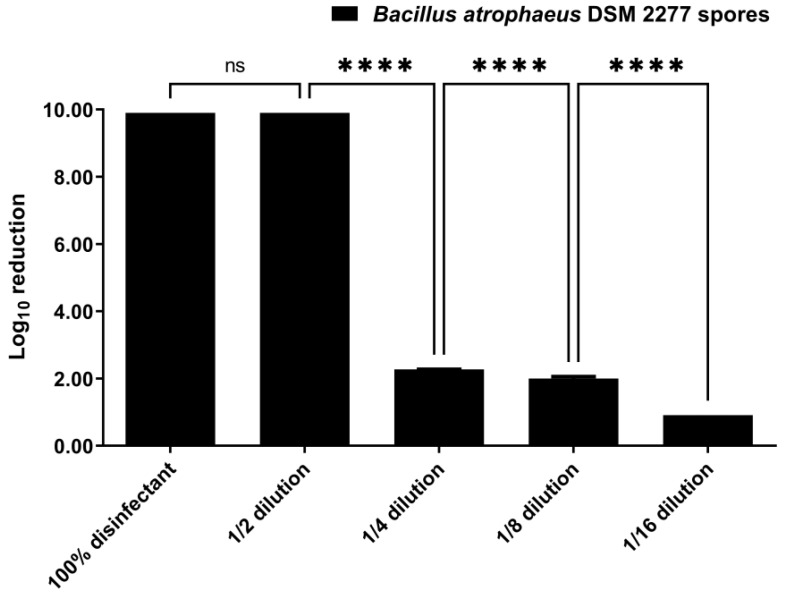
Log_10_ reduction achieved in disinfection of *Bacillus atrophaeus* DSM 2277 spores by suspension test with different disinfectant concentrations. VIRCOV BAC 360 disinfectant was tested at the following dilutions: 100% disinfectant; 1/2 (*v*/*v*) dilution; 1/4 (*v*/*v*) dilution; 1/8 (*v*/*v*) dilution; 1/16 (*v*/*v*) dilution. The contact time was 2 min. The error bars in the graph represent the standard deviation of the mean of three replicates (*n* = 3). Statistical difference between groups was evaluated by one-way analysis of variance (ANOVA) with post hoc comparisons made by the Tukey test. ns: not significant with *p*-value > 0.05; ****: significant with *p*-value < 0.0001.

Disinfection of *B. atrophaeus* DSM 2277 spores with VIRCOV BAC 360 by suspension test is shown in Figure 1. With disinfectant at both 100% concentration and 1/2 (*v*/*v*) dilution, the *B. atrophaeus* DSM 2277 spores log_10_ reduction and elimination rate were, respectively, 9.90 ± 0 and 100% (Figure 1). Further disinfectant dilutions (1/4 (*v*/*v*), 1/8 (*v*/*v*), and 1/16 (*v*/*v*) dilution) significantly reduced the number of viable spores (*p*-value < 0.0001) (Supplementary Materials Figure S5), reaching a log_10_ reduction of 2.27 ± 0.01, 2.00 ± 0.05, and 0.91 ± 0 for 1/4 (*v*/*v*), 1/8 (*v*/*v*), and 1/16 (*v*/*v*) dilutions, respectively (Figure 1). These log_10_ reductions corresponded to elimination rates of, respectively, 99.5%, 99.0%, and 87.6%. Minimum bactericidal concentration (MBC) is defined as the minimum concentration of an antimicrobial agent required to eradicate 99.9% of the microorganism isolates under testing by culturing in an antimicrobial agent-free medium [58,59]. Therefore, in accordance with the elimination rates obtained for the various dilutions, the MBC of VIRCOV BAC 360 for *B. atrophaeus* DSM 2277 spores with a contact time of 2 min is 50% of its concentration. For bacteriophage *Lambda* DSM 4499, the elimination rates for the various dilutions of VIRCOV BAC 360 were not possible to obtain because the host *E. coli* DSM 4230 was sensitive to the residual disinfectant carried in the bacteriophage suspension.

### 3.2. Disinfection by Nebulization in a 0.08 m^3^ Closet

Disinfection by nebulization of fabric swatches, a folded cotton towel, and a footwear item was tested in a 0.08 m^3^ closet.

**Figure 2:**
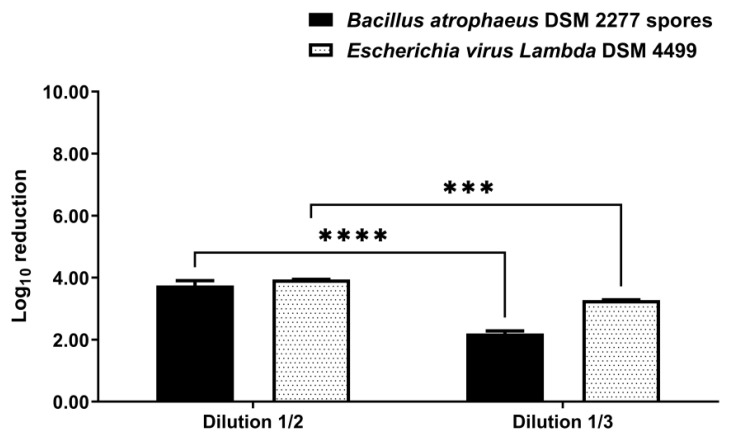
Log_10_ reduction achieved in disinfection of fabric by nebulization in a 0.08 m^3^ closet. VIRCOV BAC 360 disinfectant was tested at 1/2 (*v*/*v*) and 1/3 (*v*/*v*) dilutions. The indicators tested were *B. atrophaeus* DSM 2277 spores and bacteriophage *Escherichia virus Lambda* DSM 4499. The disinfection time consisted of 2 min of disinfectant nebulization plus 5 min of rest without nebulization. The error bars in the graph represent the standard deviation of the mean of two replicates (*n* = 2) for *B. atrophaeus* DSM 2277 spores tests and three replicates (*n* = 3) for bacteriophage *Lambda* DSM 4499 tests. Statistical difference between groups was evaluated by two-way ANOVA with post hoc comparisons made by the Tukey test. ***: significant with *p*-value < 0.001; ****: significant with *p*-value < 0.0001.

Fabric swatch disinfection was tested with nebulization of VIRCOV BAC 360 at 1/2 (*v*/*v*) dilution, which corresponds to the MBC found by the suspension test, and 1/3 (*v*/*v*) dilution, which, according to the disinfectant manufacturer, is a dilution that should continue to be effective. Both disinfectant dilutions allowed a significant reduction in the number of viable spores and bacteriophages (*p*-value < 0.0001) (Supplementary Materials Figure S6). Fabric swatches nebulization with the 1/2 (*v*/*v*) disinfectant dilution resulted in a log_10_ reduction of 3.75 ± 0.16 (99.98% elimination rate) and 3.94 ± 0.01 (99.989% elimination rate) for *B. atrophaeus* DSM 2277 spores and bacteriophage *Lambda* DSM 4499, respectively (Figure 2). For its part, nebulization with the 1/3 (*v*/*v*) disinfectant dilution resulted in a log_10_ reduction of 2.20 ± 0.09 (99.4% elimination rate) and 3.28 ± 0.01 (99.95% elimination rate) for *B. atrophaeus* DSM 2277 spores and bacteriophage *Lambda* DSM 4499, respectively (Figure 2). The 1/3 (*v*/*v*) disinfectant dilution yielded a significantly lower log_10_ reduction than the 1/2 (*v*/*v*) disinfectant dilution for both spores (*p*-value < 0.0001) and bacteriophages (*p*-value < 0.001) (Figure 2). In the spore tests, the log_10_ reduction with the 1/3 (*v*/*v*) disinfectant dilution was 1.55 log_10_ lower than that with the 1/2 (*v*/*v*) disinfectant dilution, and, in the bacteriophage tests, the log_10_ reduction with the 1/3 (*v*/*v*) disinfectant dilution was 0.66 log_10_ lower than that with the 1/2 (*v*/*v*) disinfectant dilution.

**Figure 3:**
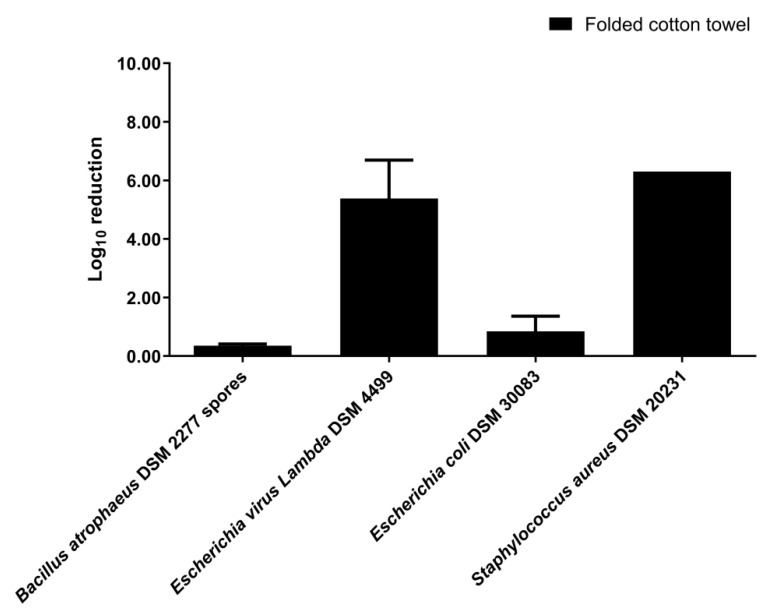
Log_10_ reduction achieved in disinfection of a folded cotton towel of 460 × 720 mm by nebulization in a 0.08 m^3^ closet. VIRCOV BAC 360 disinfectant was used at 1/3 (*v*/*v*) dilution. The indicators tested were *B. atrophaeus* DSM 2277 spores, bacteriophage *Lambda* DSM 4499, *Escherichia coli* DSM 30083, and *Staphylococcus aureus* DSM 20231. The disinfection time consisted of 2 min of disinfectant nebulization plus 5 min of rest without nebulization. The error bars in the graph represent the standard deviation of the mean of three replicates (*n* = 3).

For the folded cotton towel disinfection, inoculated fabric swatches were placed inside the folded towel (Supplementary Materials Figure S4), and then the towel was disinfected by nebulization of disinfectant at 1/3 (*v*/*v*) dilution. The disinfection by nebulization of the folded cotton towel significantly reduced the number of viable *Lambda* DSM 4499 bacteriophages (*p*-value < 0.0001) and *S. aureus* DSM 20231 cells (*p*-value < 0.0001) (Supplementary Materials Figure S7). The log_10_ reduction of bacteriophage *Lambda* DSM 4499 and *S. aureus* DSM 20231 was 5.38 ± 1.31 (99.998% elimination rate) and 6.30 ± 0.001 (100% elimination rate), respectively (Figure 3). However, for *B. atrophaeus* DSM 2277 spores and *E. coli* DSM 30083, disinfection by nebulization did not significantly reduce the number of viable spores (*p*-value > 0.05) and cells (*p*-value > 0.05) (Supplementary Materials Figure S7).

**Figure 4:**
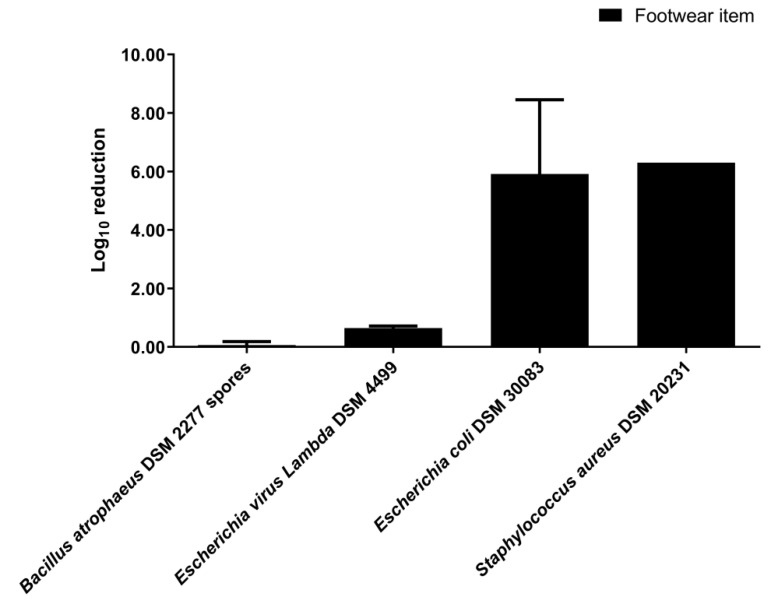
Log_10_ reduction achieved in disinfection of a footwear item by nebulization in a 0.08 m^3^ closet. VIRCOV BAC 360 disinfectant was used at 1/3 (*v*/*v*) dilution. The indicators tested were *B. atrophaeus* DSM 2277 spores, bacteriophage *Lambda* DSM 4499, *E. coli* DSM 30083, and *S. aureus* DSM 20231. The disinfection time consisted of 2 min of disinfectant nebulization plus 5 min of rest without nebulization. The error bars in the graph represent the standard deviation of the mean of three replicates (*n* = 3).

The disinfection by nebulization of the footwear item significantly reduced the number of viable *E. coli* DSM 30083 (*p*-value < 0.0001) and *S. aureus* DSM 20231 cells (*p*-value < 0.0001) (Supplementary Materials Figure S8). The log_10_ reductions of *E. coli* DSM 30083 and *S. aureus* DSM 20231 were 5.91 ± 2.55 (99.96% elimination rate) and 6.30 ± 0.001 (100% elimination rate), respectively (Figure 4). However, for *B. atrophaeus* DSM 2277 spores and bacteriophage *Lambda* DSM 4499, disinfection by nebulization in the 0.08 m^3^ closet did not significantly reduce their numbers (*p*-value > 0.05 for both) (Supplementary Materials Figure S8).

### 3.3. Disinfection by Nebulization in a 0.58 m^3^ Closet

**Figure 5:**
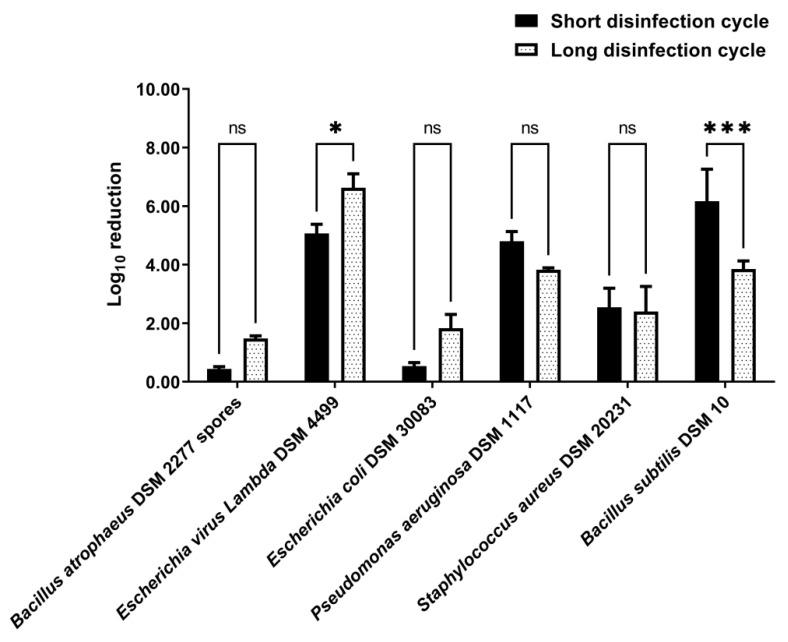
Log_10_ reduction achieved in disinfection of fabric by nebulization in the upscaled 0.58 m^3^ closet. VIRCOV BAC 360 disinfectant was used at a 1/3 (*v*/*v*) dilution. The indicators tested were *B. atrophaeus* DSM 2277 spores, bacteriophage *Lambda* DSM 4499, *E. coli* DSM 30083, *Pseudomonas aeruginosa* DSM 1117, *S. aureus* DSM 20231, and *Bacillus subtilis* DSM 10. For the disinfection time, a short disinfection cycle and a long disinfection cycle were tested. The short disinfection cycle consisted of 2 min of nebulization, 2 min of rest without nebulization, and 2 min of aerosol extraction from the closet interior. For its part, the long disinfection cycle consisted of 4 min of nebulization, 4 min of rest without nebulization, and 2 min of aerosol extraction from the closet interior. The error bars in the graph represent the standard deviation of the mean of three replicates (*n* = 3). Statistical difference between groups was evaluated by two-way ANOVA with post hoc comparisons made by the Tukey test. ns: not significant with *p*-value > 0.05; *: significant with *p*-value < 0.05; ***: significant with *p*-value < 0.001.

Disinfection by nebulization of fabric swatches was also tested in the upscaled 0.58 m^3^ closet with a short disinfection cycle and a long disinfection cycle. The use of a short disinfection cycle significantly reduced the number of viable *Lambda* DSM 4499 bacteriophages (*p*-value < 0.0001) and the number of viable *P. aeruginosa* DSM 1117, *S. aureus* DSM 20231, and *B. subtilis* DSM 10 cells (*p*-value < 0.0001) inoculated in the fabric swatches (Supplementary Materials Figure S9). However, the short disinfection cycle did not significantly reduce the number of viable *B. atrophaeus* DSM 2277 spores (*p*-value > 0.05) and *E. coli* DSM 30083 cells (*p*-value > 0.05) (Supplementary Materials Figure S9). In the short disinfection cycle experiments, the log_10_ reductions of bacteriophage *Lambda* DSM 4499, *P. aeruginosa* DSM 1117, *S. aureus* DSM 20231, and *B. subtilis* DSM 10 were 5.07 ± 0.31 (99.999% elimination rate), 4.80 ± 0.33 (99.999% elimination rate), 2.54 ± 0.66 (99.4% elimination rate), and 6.17 ± 1.09 (99.9997% elimination rate), respectively (Figure 5). For its part, the use of a long disinfection cycle significantly reduced the number of viable microorganisms inoculated in the fabric swatches for all indicators tested (*p*-value < 0.01) (Supplementary Materials Figure S10). In the long disinfection cycle experiments, the log_10_ reductions of *B. atrophaeus* DSM 2277 spores, bacteriophage *Lambda* DSM 4499, *E. coli* DSM 30083, *P. aeruginosa* DSM 1117, *S. aureus* DSM 20231, and *B. subtilis* DSM 10 were 1.48 ± 0.09 (96.7% elimination rate), 6.63 ± 0.48 (99.99997% elimination rate), 1.83 ± 0.47 (98.2% elimination rate), 3.83 ± 0.07 (99.99% elimination rate), 2.40 ± 0.86 (99.4% elimination rate), and 3.85 ± 0.28 (99.98% elimination rate), respectively (Figure 5). For the indicators *B. atrophaeus* DSM 2277 spores, *E. coli* DSM 30083, *P. aeruginosa* DSM 1117, and *S. aureus* DSM 20231, the log_10_ reductions of the experiments with a short disinfection cycle was not significantly different from the log_10_ reduction of the experiments with a long disinfection cycle (*p*-value > 0.05) (Figure 5). However, for bacteriophage *Lambda* DSM 4499, the log_10_ reduction of the long disinfection cycle was significantly greater than the log_10_ reduction of the short disinfection cycle (*p*-value < 0.05), with the log_10_ reduction of the long disinfection cycle being 1.56 log_10_ greater than the log_10_ reduction of the short disinfection cycle (Figure 5). On the other hand, for *B. subtilis* DSM 10, the result was the opposite (*p*-value < 0.001), with the log_10_ reduction of the long disinfection cycle being 2.32 log_10_ lower than the log_10_ reduction of the short disinfection cycle (Figure 5).

## 4. Discussion

Pathogens can lodge on clothing and footwear, and lead to the spread of infections, putting public health at risk [7,8,9,10,11]. Clothing and footwear are commonly disinfected by laundering [7,8,9,10,11,23,24,25]. However, laundering is not practicable in stores where materials are shared between customers. Therefore, the availability of an automatic, fast, and effective disinfection system is relevant.

In the present study, the disinfection of textiles and footwear in an ultrasonic nebulizer closet was evaluated by the use of several indicators. *B. atrophaeus* spores are commonly used as indicators of sterilization and biocidal activity of chemical agents [60], and here they were used as an indicator of chemical sterilization efficiency. In the context of the COVID-19 pandemic, the virucidal capacity of disinfection systems has a further interest. SARS-CoV-2 is reported in the literature as being sensitive to the biocidal active substances present in VIRCOV BAC 360 [61,62,63,64,65,66,67,68,69]. Since the bacteriophage *Lambda* DSM 4499, used in the present study as a virucidal indicator, is non-enveloped [70], it is considered less susceptible to disinfection with this disinfectant than enveloped SARS-CoV-2 [71,72,73]. Therefore, the bacteriophage elimination suggests a possible action of VIRCOV BAC 360 on SARS-CoV-2. However, as pointed out by Nims and Zhou [74], caution should be taken when viral inactivation susceptibilities are extrapolated from one virus to another. In the present study, several species of bacteria were also used as indicators of bactericidal activity. Thus, vegetative cells of *E. coli* DSM 30083 and *P. aeruginosa* DSM 1117 were used as indicators of Gram-negative bactericidal activity, and vegetative cells of *S. aureus* DSM 20231 and *B. subtilis* DSM 10 were used as indicators of Gram-positive bactericidal activity.

In the present study, the disinfectant VIRCOV BAC 360 in the suspension tests with *B. atrophaeus* DSM 2277 spores exceeded the 5 log_10_ reduction required by EN 13727 and EN 1276 standards [47] when applied at 100% concentration and at 1/2 dilution, but not to other dilutions. Nonetheless, it should be noted that these standards refer to the elimination of vegetative bacteria [47], not bacterial spores, as was the case of the suspension tests of the present study. Bacterial spores are substantially more resistant to disinfection than vegetative bacterial cells [73,75]. Thus, here, the EN 13704 standard [55], which provides a quantitative suspension test for the evaluation of the bacterial sporicidal activity of chemical disinfectants used in food, industrial, domestic, and institutional sectors [56], was also considered. Following this standard, the disinfectant VIRCOV BAC 360 in the suspension test with *B. atrophaeus* DSM 2277 spores also reached the pass criterion (3 log_10_ reduction) when it was applied at 100% concentration and at 1/2 dilution.

The nebulization disinfection experiments of the present study did not reach the 7 log_10_ reduction of bacteria indicators required by the EN 16616 standard as a pass criterion [47]. However, according to ASTM E2406 and ASTM E2274 standards (4 log_10_ reduction as a pass criterion [47]), the disinfection process was validated for the following conditions and bacteria indicators: (1) folded cotton towel disinfection in the 0.08 m^3^ closet (*S. aureus* DSM 20231); (2) footwear item disinfection in the 0.08 m^3^ closet (*E. coli* DSM 30083 and *S. aureus* DSM 20231); (3) fabric disinfection with a short disinfection cycle in the 0.58 m^3^ closet (*P. aeruginosa* DSM 1117 and *B. subtilis* DSM 10). These three standards do not define pass criteria for viruses [47]. Therefore, considering the same criteria as the ones of the ASTM E2406 and ASTM E2274 standards adopted above for bacteria indicators [47], the disinfection process was effective for the bacteriophage in all nebulization experiments, except for the fabric and the footwear item experiments in the 0.08 m^3^ closet. The EN 16616 [52], ASTM E2406 [53], and ASTM E2274 [54] standards are directed to processes conducted in washing machines [7]. Hence, the fact of the nebulization experiments of the present study have reached the pass criteria of ASTM E2406 and ASTM E2274 standards [47] is remarkable and shows that disinfection of clothing and footwear in an ultrasonic nebulization closet can be as effective as in a washing machine.

In view of the U.S. Pharmacopeia pass criteria (2 log_10_ reduction for bacterial spores and 3 log_10_ reduction for vegetative bacteria) [57], the disinfection process was effective for the following conditions and indicators: (1) fabric disinfection with the 0.08 m^3^ closet using both 1/2 (*v*/*v*) and 1/3 (*v*/*v*) disinfectant dilution (*B. atrophaeus* DSM 2277 spores); (2) folded cotton towel disinfection with the 0.08 m^3^ closet (*S. aureus* DSM 20231); (3) footwear item disinfection with the 0.08 m^3^ closet (*E. coli* DSM 30083 and *S. aureus* DSM 20231); (4) fabric disinfection with both short and long disinfection cycle in the 0.58 m^3^ closet (*P. aeruginosa* DSM 1117 and *B. subtilis* DSM 10). Therefore, according to the U.S. Pharmacopeia [57], the disinfection of clothing and footwear in an ultrasonic nebulization closet is attained.

As noted by Zonta et al. [76] and Callahan et al. [39], the comparison of disinfection results between different studies is difficult because of differences between disinfectants, carrier materials, application methods and conditions, and indicator strains. Nevertheless, as in the present study, Callahan et al. [39] also developed a disinfection chamber for the application of a nebulized disinfectant. The log_10_ reductions obtained in the 0.58 m^3^ closet for the disinfection of *S. aureus* DSM 20231 on fabric are in line with the log_10_ reductions obtained by Callahan et al. [39] for methicillin-resistant *S. aureus* (MRSA) on both carpet and fabric. Furthermore, the log_10_ reductions obtained in the 0.58 m^3^ closet for the disinfection of *B. subtilis* DSM 10 were higher than those obtained by these authors for the Gram-positive indicators (MRSA and vancomycin-resistant *Enterococci*) used on both carpet and fabric. Additionally, in the 0.58 m^3^ closet, the 6 min disinfection time of the short disinfection cycle experiment was considerably shorter than the 1 h disinfection time of the system used by Callahan et al. [39]. Therefore, the present system can be applied for the quick disinfection of clothing and footwear in stores after customer handling.

Clothing and footwear disinfection in an ultrasonic nebulization closet such as the 0.58 m^3^ closet presents advantages, such as quick disinfection without wetting the materials; application of disinfectant in the aerosol form, which allows the disinfectant to penetrate porous surfaces, such as clothing; reaching all parts of an irregular surface, such as shoes; disinfection of clothes and footwear that cannot withstand high temperatures or cannot be washed, and it can be performed at room temperature. Moreover, during operation, the 0.58 m^3^ closet can be operated in the presence of people since it is completely sealed, and, at the end of the disinfection cycle, the aerosol present inside the closet is extracted through activated carbon filters.

The disinfectant used in the present study also has advantages, namely, it does not stain or damage clothes or footwear and the quaternary ammonium compound included in its formulation interacts with the surface of negatively charged textiles [7].

Some limitations to the disinfection assessment can be pointed out, such as the non-use of human viruses, the non-use of unclean materials, and the non-use of a neutralizer to inactivate the residual disinfectant. Nevertheless, in the nebulization experiments, the effect of the residual disinfectant was not expected because the indicators were removed from the closet after disinfection, being no longer in contact with the aerosol. Additionally, in the nebulization experiments, the indicators were placed immediately into sterile 0.85% (*w*/*v*) NaCl after disinfection, which diluted the residual disinfectant.

## 5. Conclusions

Finding an efficient method for the quick disinfection of textiles and footwear can be decisive for controlling the spread of infections. The disinfection of textiles and footwear by disinfectant aerosolization in an ultrasonic nebulizer closet is an automated easy-to-use alternative. The results of the present study show that, in a 0.08 m^3^ closet, the ultrasonic nebulization of the disinfectant used in this work at a 1/3 dilution allowed efficient disinfection of fabric inoculated with *B. atrophaeus* DSM 2277 spores in 7 min. In the same 0.08 m^3^ closet and under the same conditions, the ultrasonic nebulization disinfection of a folded cotton towel was achieved for bacteriophage *Lambda* DSM 4499 and *S. aureus* DSM 20231. Similarly, the disinfection of a footwear item was also achieved for *E. coli* DSM 30083 and *S. aureus* DSM 20231 in the 0.08 m^3^ closet under the same conditions. In the upscaled 0.58 m^3^ closet, the ultrasonic nebulization of the disinfectant at 1/3 dilution allowed efficient disinfection of fabric inoculated with bacteriophage *Lambda* DSM 4499, *P. aeruginosa* DSM 1117, and *B. subtilis* DSM 10 in 6 min. Disinfection by ultrasonic nebulization in a closet was shown to be as effective as laundering methods, with the advantage of not wetting the materials and being much faster. Furthermore, the ultrasonic nebulization provides a very fine aerosol that penetrates the pores of the fabric and reaches all parts of footwear. In addition, a sealed closet equipped with aerosol exhaust with activated carbon filters, such as the 0.58 m^3^ closet used in the present study, can be operated in the presence of people. Thus, ultrasonic nebulization disinfection in a closet system proves to be useful in clothing and footwear stores to prevent pathogen transmission by the items’ widespread handling.

## Figures and Tables

**Table 1:** Incubation conditions for bacteria cultivation in agar medium plates.

Bacterium	Agar Growing Medium	Incubation Temperature (°C)	Incubation Time (h)
*Bacillus atrophaeus* DSM 2277	NA ^1^	30	48
*Escherichia coli* DSM 4230	LB agar ^2^	37	24
*Escherichia coli* DSM 30083	NA ^1^	37	24
*Pseudomonas aeruginosa* DSM 1117	NA ^1^	37	24
*Staphylococcus aureus* DSM 20231	NA ^1^	37	48
*Bacillus subtilis* DSM 10	NA ^1^	30	24

^1^ NA: nutrient agar medium. ^2^ LB agar: Luria–Bertani agar medium.

## Data Availability

The data reported in the present study are available within this article and in the Supplementary Materials.

## Supplementary Materials

The following are available online at https://www.mdpi.com/article/10.3390/ijerph191710472/s1, Figure S1: Closet of 0.08 m^3^ with the following dimensions: 605 mm (height) × 420 mm (width) × 325 mm (length); Figure S2: Closet of 0.58 m^3^ with the following dimensions: 1600 mm (height) × 600 mm (width) × 600 mm (length); Figure S3: Example of fabric swatches of approximately 6 cm^2^ composed of 88% polyester and 12% elastane used in nebulization disinfection experiments; Figure S4: Example of the preparation of the folded cotton towel of 460 × 720 mm for nebulization disinfection experiments in the 0.08 m^3^ closet; Figure S5: Log_10_(CFU·mL^−1^) obtained in disinfection of *Bacillus atrophaeus* DSM 2277 spores by suspension test with different disinfectant concentrations; Figure S6: Log_10_(CFU or PFU·mL^−1^) obtained in control and disinfection tests of disinfection of fabric by nebulization in a 0.08 m^3^ closet; Figure S7: Log_10_(CFU or PFU·mL^−1^) obtained in control and disinfection tests of disinfection of a folded cotton towel of 460 × 720 mm by nebulization in a 0.08 m^3^ closet; Figure S8: Log_10_(CFU or PFU·mL^−1^) obtained in control and disinfection tests of disinfection of a footwear item by nebulization in a 0.08 m^3^ closet; Figure S9: Log_10_(CFU or PFU·mL^−1^) obtained in control and disinfection tests of disinfection of fabric by nebulization in the upscaled 0.58 m^3^ closet by employing a short disinfection cycle; Figure S10: Log_10_(CFU or PFU·mL^−1^) obtained in control and disinfection tests of disinfection of fabric by nebulization in the upscaled 0.58 m^3^ closet by employing a long disinfection cycle.
