# Supplementary material for: Application of an Ultrasonic Nebulizer Closet in the Disinfection of Textiles and Footwear
Source: Int J Environ Res Public Health. 2022 Aug 23;19(17):10472. doi: 10.3390/ijerph191710472 (PMC9518335; doi:10.3390/ijerph191710472)
Supplement: Supplementary file 1 [file ijerph-19-10472-s001.zip › ijerph-1827494-supplementary.pdf]

International Journal of
Environmental Research and Public Health

MDPI

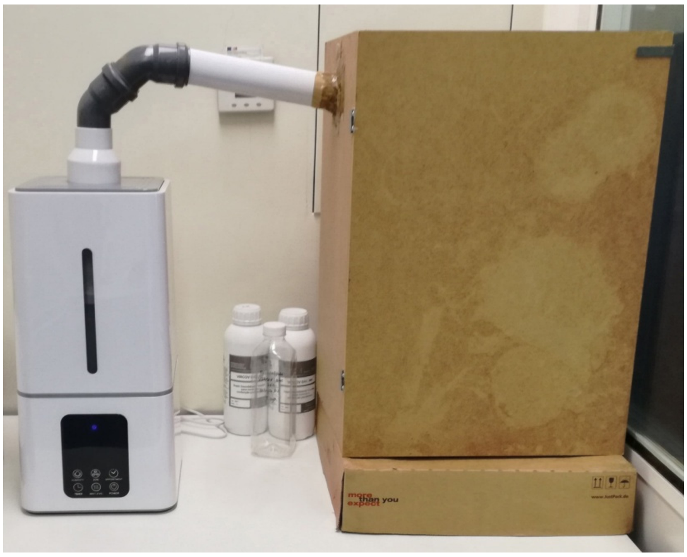

Figure S1. Closet of 0.08 m^3 with the following dimensions: 605 mm (height) × 420 mm (width) × 325 mm (length). The closet was equipped with a piezoelectric ultrasonic nebulizer to aerosolize the disinfectant solution into the closet chamber.

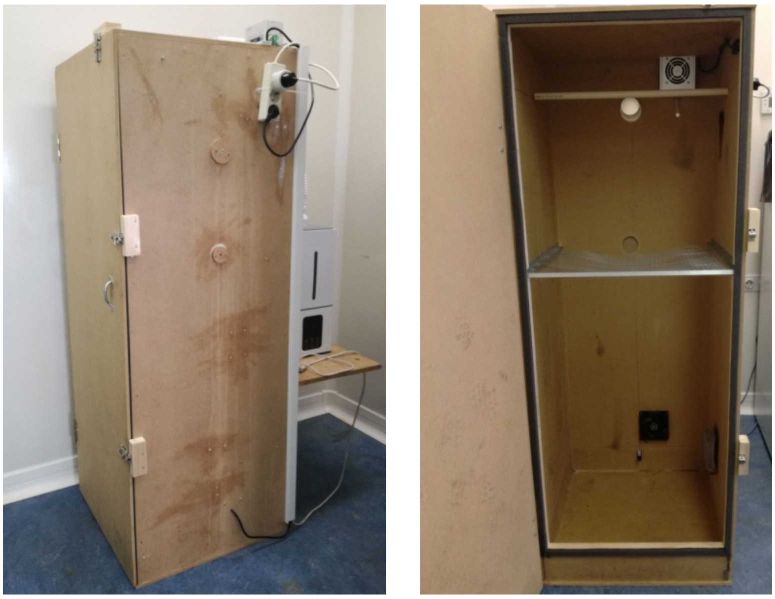

(**a**) (**b**)

Figure S2. Closet of 0.58 m^3 with the following dimensions: 1600 mm (height) × 600 mm (width) × 600 mm (length). The 0.58 m^3 closet was a prototype of NovirBox from Dynasys – Engenharia e Telecomunicações, S.A., Portugal, and was equipped with a piezoelectric ultrasonic nebulizer to aerosolize the disinfectant solution into the closet chamber. The closet was also equipped with a dehumidifier, a heater, and an exhaust fan with activated carbon filters. (**a**) Exterior of the 0.58 m^3 closet. (b) Interior of the 0.58 m^3 closet.

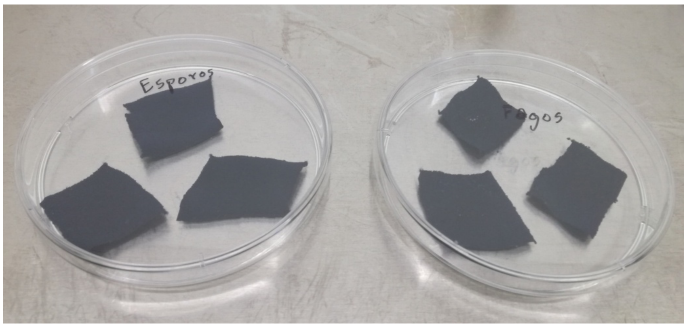

Figure S3. Example of fabric swatches of approximately 6 cm² composed of 88% polyester and 12% elastane used in nebulization disinfection experiments.

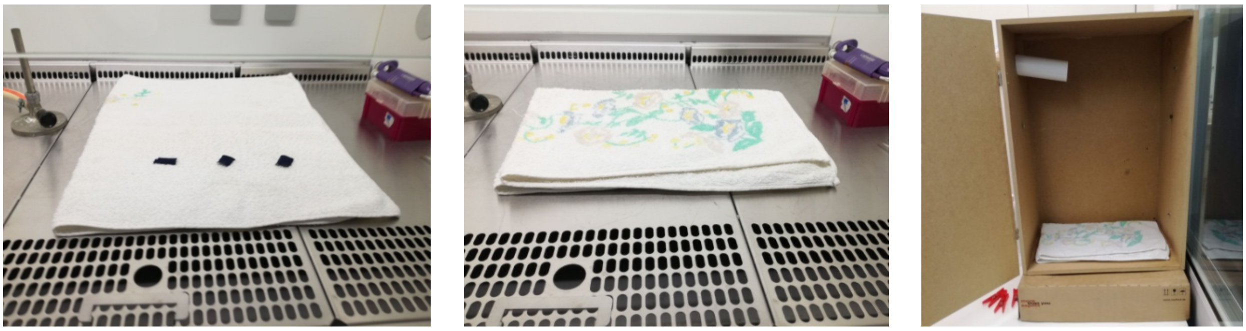

(**a**) (**b**) (**c**)

Figure S4. Example of the preparation of the folded cotton towel of 460 × 720 mm for nebulization disinfection experiments in the 0.08 m³ closet. For the folded cotton towel disinfection experiments, first, fabric swatches of approximately 6 cm² composed of 88% polyester and 12% elastane were inoculated with 30 µL of indicator suspension to be tested. Then, after the 30 µL of inoculum had been absorbed by fabric, the swatches were transferred onto the folded cotton towel. Subsequently, the towel was folded again to cover the fabric swatches and then placed at the bottom of the 0.08 m³ closet chamber. (**a**) Inoculated fabric swatches over the folded cotton towel. (**b**) Cotton towel folded over the fabric swatches. (**c**) Folded cotton towel, with the inoculated fabric swatches inside, placed at the bottom of the 0.08 m³ closet chamber.

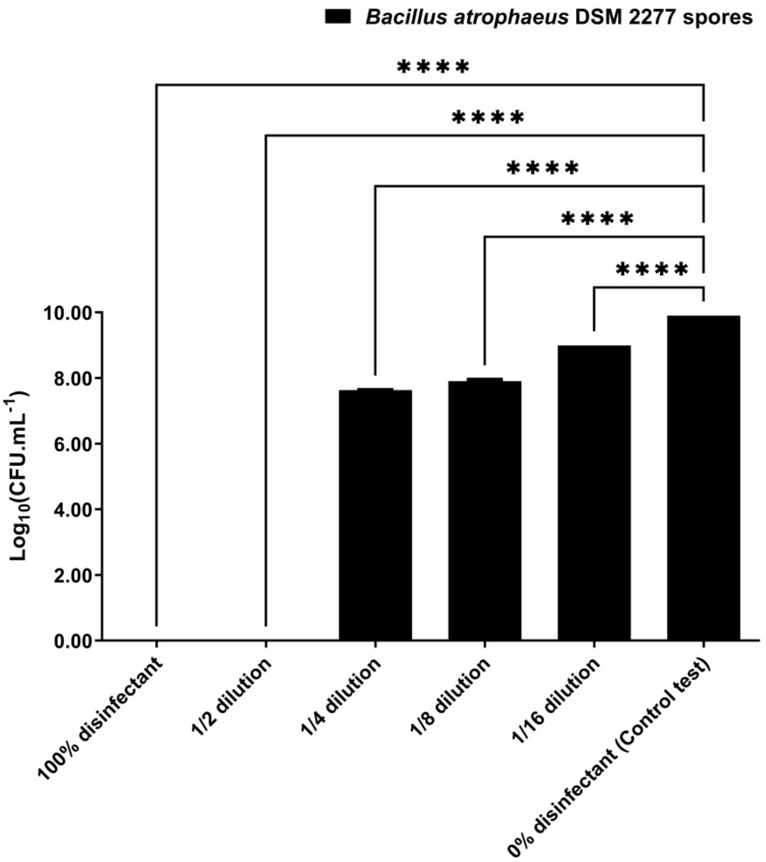

Figure S5. $Log_{10}(CFU{\cdot}mL^{-1})$ obtained in disinfection of *Bacillus atrophaeus* DSM 2277 spores by suspension test with different disinfectant concentrations. VIRCOV BAC 360 disinfectant was tested at the following dilutions: 100% disinfectant; 1/2 (v/v) dilution; 1/4 (v/v) dilution; 1/8 (v/v) dilution; 1/16 (v/v) dilution and 0% disinfectant (control test). The test with 0% disinfectant, which represented the control test, consisted of sterile deionized water only. The contact time was 2 min. With the exception of the 0% disinfectant test (control test), which only consisted of one replicate (n=1), the remaining error bars in the graph represent the standard deviation of the mean of three replicates (n=3). Statistical difference between groups was evaluated by one-way analysis of variance (ANOVA) with post hoc comparisons made by the Tukey test. ****: significant with p-value < 0.0001.

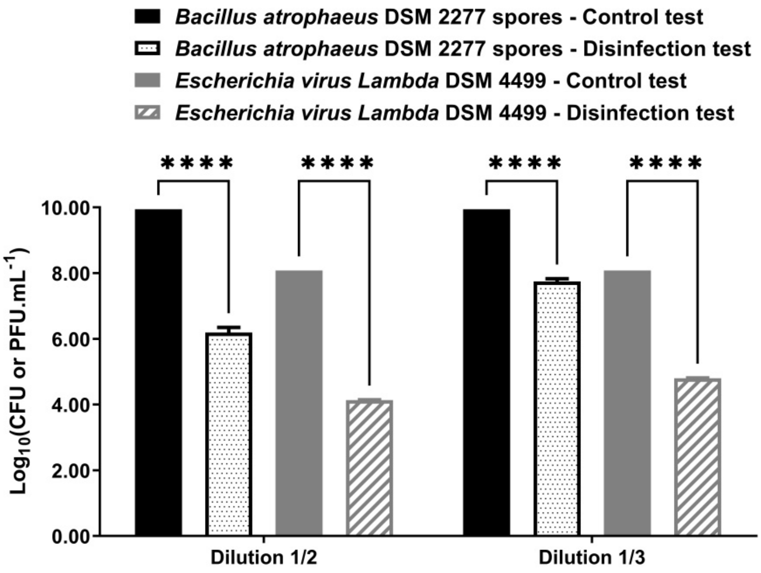

Figure S6. Log$_{10}$(CFU or PFU·mL^{-1}) obtained in control and disinfection tests of disinfection of fabric by nebulization in a 0.08 m^3 closet. VIRCOV BAC 360 disinfectant was tested at 1/2 (v/v) and 1/3 (v/v) dilutions. The indicators tested were *B. atrophaeus* DSM 2277 spores and bacteriophage *Escherichia virus Lambda* DSM 4499. The disinfection time consisted of 2 min of disinfectant nebulization plus 5 min of rest without nebulization. In control tests, the indicators were not subjected to the disinfection exposure step. The error bars in the graph represent the standard deviation of the mean of two replicates (n=2) for *B. atrophaeus* DSM 2277 spores disinfection tests and three replicates (n=3) for bacteriophage *Lambda* DSM 4499 disinfection tests. The control tests, for their part, consisted of only a single replicate (n=1). Statistical difference between groups was evaluated by two-way ANOVA with post hoc comparisons made by the Tukey test. ****: significant with p-value < 0.0001.

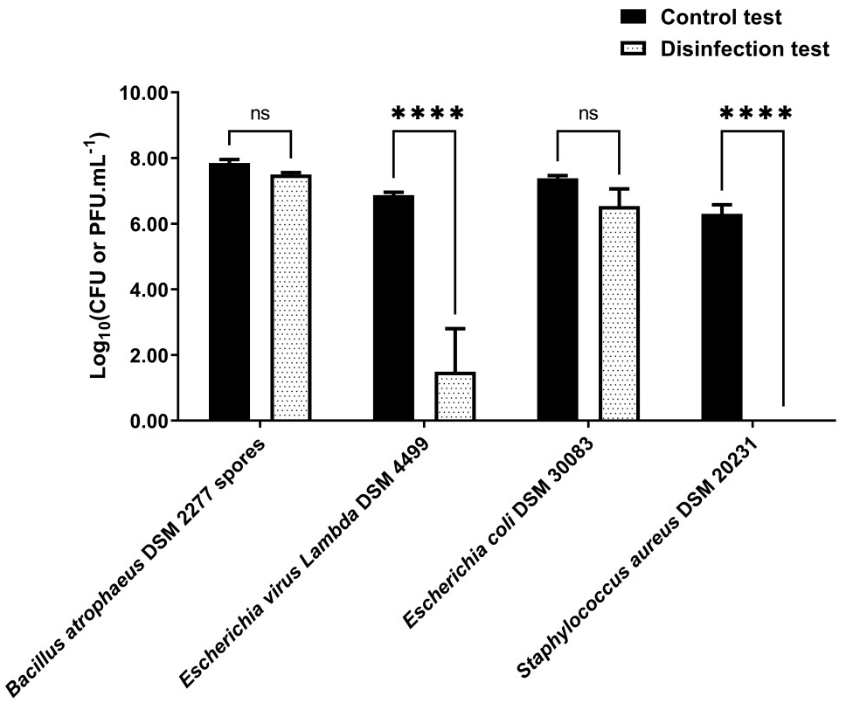

Figure S7. Log_{10}(CFU or PFU·mL^{-1}) obtained in control and disinfection tests of disinfection of a folded cotton towel of 460 × 720 mm by nebulization in a 0.08 m^3 closet. VIRCOV BAC 360 disinfectant was used at 1/3 (v/v) dilution. The indicators tested were *B. atrophaeus* DSM 2277 spores, bacteriophage *Lambda* DSM 4499, *Escherichia coli* DSM 30083, and *Staphylococcus aureus* DSM 20231. The disinfection time consisted of 2 min of disinfectant nebulization plus 5 min of rest without nebulization. In control tests, the indicators were not subjected to the disinfection exposure step. The error bars in the graph represent the standard deviation of the mean of three replicates (n=3) in both control and disinfection tests. Statistical difference between groups was evaluated by two-way ANOVA with post hoc comparisons made by the Tukey test. ns: not significant with p-value > 0.05; ****: significant with p-value < 0.0001.

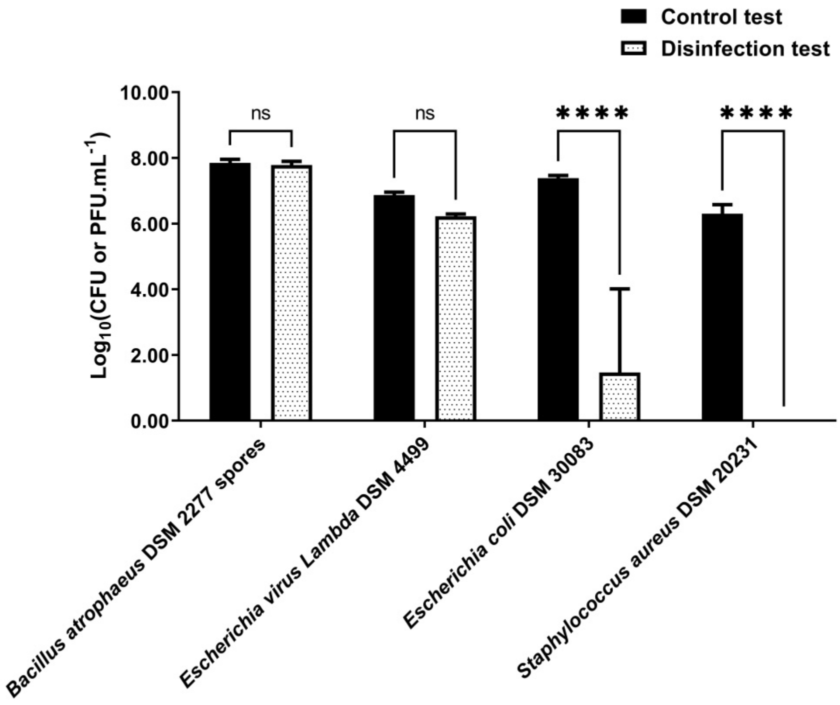

Figure S8. Log_{10}(CFU or PFU·mL^{-1}) obtained in control and disinfection tests of disinfection of a footwear item by nebulization in a 0.08 m^3 closet. VIRCOV BAC 360 disinfectant was used at 1/3 (v/v) dilution. The indicators tested were *B. atrophaeus* DSM 2277 spores, bacteriophage *Lambda* DSM 4499, *E. coli* DSM 30083, and *S. aureus* DSM 20231. The disinfection time consisted of 2 min of disinfectant nebulization plus 5 min of rest without nebulization. In control tests, the indicators were not subjected to the disinfection exposure step. The error bars in the graph represent the standard deviation of the mean of three replicates (n=3) in both control and disinfection tests. Statistical difference between groups was evaluated by two-way ANOVA with post hoc comparisons made by the Tukey test. ns: not significant with p-value > 0.05; ****: significant with p-value < 0.0001.

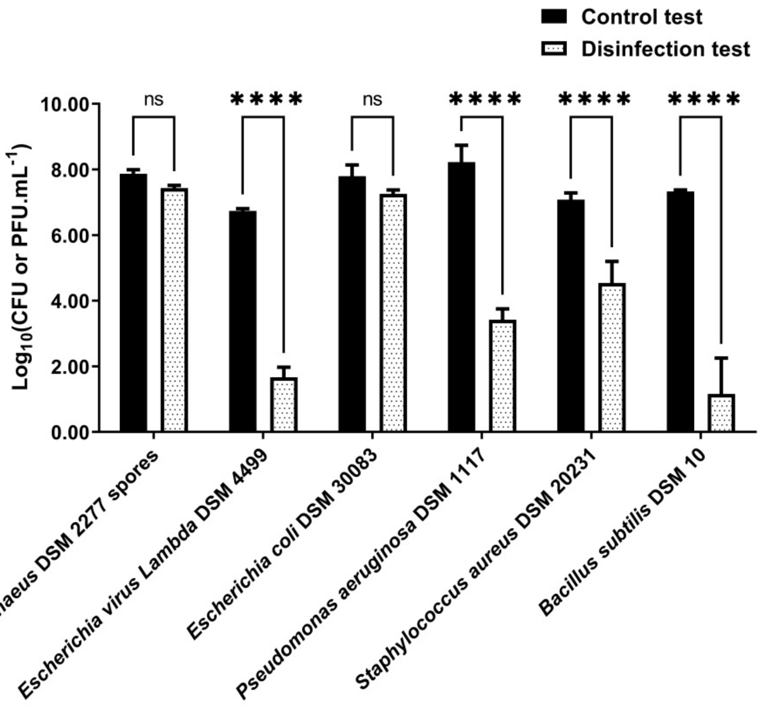

Figure S9. Log_{10}(CFU or PFU·mL^{-1}) obtained in control and disinfection tests of disinfection of fabric by nebulization in the upscaled 0.58 m^3 closet by employing a short disinfection cycle. VIRCOV BAC 360 disinfectant was used at 1/3 (v/v) dilution. The indicators tested were *B. atrophaeus* DSM 2277 spores, bacteriophage *Lambda* DSM 4499, *E. coli* DSM 30083, *Pseudomonas aeruginosa* DSM 1117, *S. aureus* DSM 20231, and *Bacillus subtilis* DSM 10. The short disinfection cycle consisted of 2 min of nebulization, 2 min of rest without nebulization, and 2 min of aerosol extraction from the closet interior. In control tests, the indicators were not subjected to the disinfection exposure step. The error bars in the graph represent the standard deviation of the mean of three replicates (n=3) in both control and disinfection tests. Statistical difference between groups was evaluated by two-way ANOVA with post hoc comparisons made by the Tukey test. ns: not significant with p-value > 0.05; ****: significant with p-value < 0.0001.

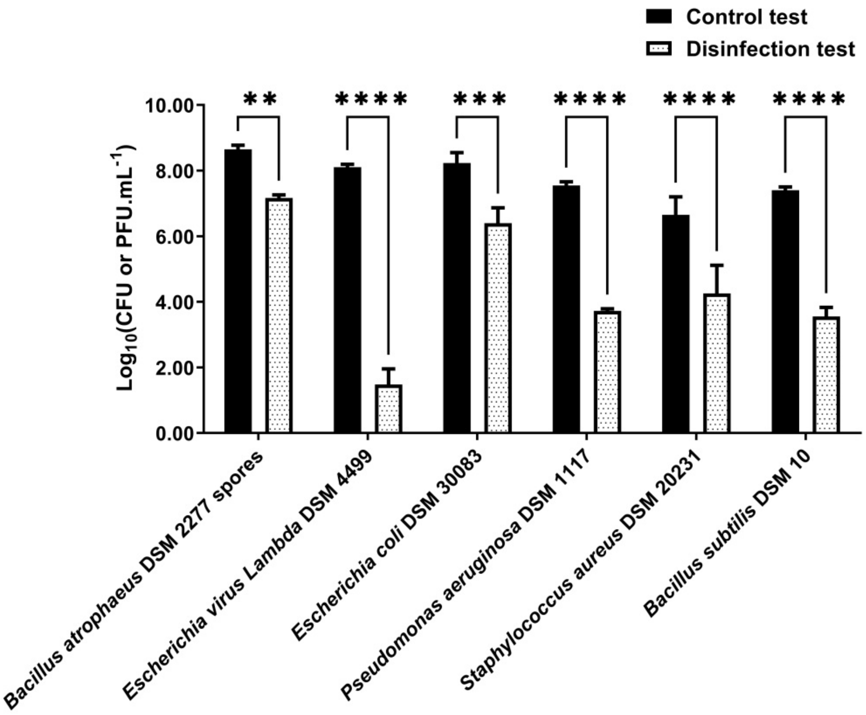

Figure S10. Log_{10}(CFU or PFU·mL^{-1}) obtained in control and disinfection tests of disinfection of fabric by nebulization in the upscaled 0.58 m^3 closet by employing a long disinfection cycle. VIRCOV BAC 360 disinfectant was used at 1/3 (v/v) dilution. The indicators tested were *B. atrophaeus* DSM 2277 spores, bacteriophage *Lambda* DSM 4499, *E. coli* DSM 30083, *P. aeruginosa* DSM 1117, *S. aureus* DSM 20231, and *B. subtilis* DSM 10. The long disinfection cycle consisted of 4 min of nebulization, 4 min of rest without nebulization, and 2 min of aerosol extraction from the closet interior. In control tests, the indicators were not subjected to the disinfection exposure step. The error bars in the graph represent the standard deviation of the mean of three replicates (n=3) in both control and disinfection tests. Statistical difference between groups was evaluated by two-way ANOVA with post hoc comparisons made by the Tukey test. **: significant with p-value < 0.01; ***: significant with p-value < 0.001; ****: significant with p-value < 0.0001.
